# GLP-1 receptor agonists and coronary plaques regression in diabetic patients after acute coronary syndromes

**DOI:** 10.1007/s00592-025-02606-z

**Published:** 2025-11-04

**Authors:** Mauro Gitto, Federica Catapano, Marco Francone, Gianluca Mincione, Vincenzo Scialò, Carlo A. Pivato, Costanza Lisi, Damiano Regazzoli, Davide Cao, Roberta Maria Fiorina, Alessandra Petrelli, Loredana Bucciarelli, Cristian Loretelli, Gianluigi Condorelli, Paolo Fiorina, Giulio Stefanini

**Affiliations:** 1 Department of Biomedical Sciences, Humanitas University, Pieve Emanuele, Milan, Italy; 2 IRCCS Humanitas Research Hospital, Rozzano, Milan, Italy; 3 International Center for T1D, Pediatric Clinical Research Center Romeo Ed Enrica Invernizzi, DIBIC, Università Degli Studi di Milano, Milan, Italy; 4 Nephrology Division, Boston Children’s Hospital, Harvard Medical School, Boston, MA USA; 5 Division of Endocrinology, ASST Fatebenefratelli-Sacco, Milan, Italy

**Keywords:** GLP-1 receptor agonists, Diabetes mellitus, Coronary artery disease, Acute coronary syndrome, Plaque regression

## Abstract

**Background:**

Despite advances in therapeutic strategies a significant proportion of acute coronary syndrome (ACS) patients experience early coronary artery disease (CAD) progression, particularly those with diabetes.

**Aim:**

To evaluate CAD progression in diabetic patients treated with glucagon-like peptide 1 receptor agonists (GLP-1Ra) over 1 year after an ACS.

**Methods:**

Patients presenting with non–ST-elevation ACS between 2019 and 2022 were enrolled in a prospective registry and underwent serial coronary computed tomography angiography (CCTA) at baseline (after revascularization, during the index hospitalization) and at 1-year follow-up. The primary endpoint was the absolute change (1 year – baseline) in non-culprit lesion plaque burden (ΔPB) on CCTA, with the absolute change in patient percent atheroma volume (ΔPAV) as a key secondary endpoint. A comprehensive lipidomic, metabolomic, and proteomic plasma assessment was also performed in all GLP-1Ra–treated patients and four randomly selected controls.

**Results:**

Of 28 diabetic patients, 7 (25%) with 22 coronary plaques were treated with GLP-1Ra, and 21 (75%) with 65 plaques received other antidiabetic agents. In the 1-year observation frame, both ΔPB (-5.8 ± 12.8% vs. -1.1 ± 13.6%, *p* = 0.041) and ΔPAV (-6.1% [-7.3, -1.8] vs. -0.7% [-2.4, 9.8], *p* = 0.039) were significantly lower in GLP-1Ra-treated patients. Total atheroma volume also showed a numerically greater reduction in the GLP-1Ra cohort (0.7 mm³ [-2.5-8.7] vs. 25.0 mm³ [4.8–39.7]), primarily due to a decrease in plaque fibrofatty volume percentage (-2.9 ± 10.1% vs. 1.0 ± 6.8%, *p* = 0.042). Lipidomic, metabolomic, and proteomic analyses identified reductions in monoacylglycerols and triacylglycerols, increases in diacylglycerols and phosphatidylethanolamine, a shift from carbohydrate metabolism toward lipid metabolism and hormone regulation, and differential expression of proteins involved in complement activation, endothelial function, and cytoskeletal organization in GLP-1Ra–treated patients compared with controls.

**Conclusions:**

In diabetic patients with ACS, GLP-1Ra therapy was associated with a significant regression in coronary plaque burden at 1 year, supported by favorable lipidomic, metabolomic, and proteomic changes. These findings suggest a potential role for GLP-1Ra in modifying atherosclerosis progression beyond glycemic control.

**Supplementary Information:**

The online version contains supplementary material available at 10.1007/s00592-025-02606-z.

## Background

Acute coronary syndrome (ACS) remains a leading cause of cardiovascular mortality globally [1]. Despite ongoing advancements in coronary revascularization and secondary prevention strategies, up to 20% of ACS patients experience recurrent myocardial infarction (MI) within five years after the index event [2–4], due to either stent failure or progression of native coronary artery disease (CAD) in non-treated segments [5]. The risk of coronary plaques progression is particularly high in diabetic patients, who exhibit a proinflammatory and prothrombotic state that accelerates atherosclerosis and increases the likelihood of recurrent ischemic events [6, 7]. Novel antidiabetic agents, including glucagon-like peptide 1 receptor agonists (GLP-1Ra) and sodium-glucose cotransporter 2 (SGLT2) inhibitors, have demonstrated cardiovascular benefits and are currently recommended in patients with type 2 diabetes mellitus (T2D) and established cardiovascular disease [8, 9]. In particular, GLP-1Ra have been shown to reduce major adverse cardiovascular events in diabetic patients with established atherosclerosis [10]. While the mechanisms underlying these benefits remain incompletely understood, plaque stabilization has been proposed as a potential contributing factor. As tailored risk stratification is crucial for optimizing secondary prevention strategies, coronary computed tomography angiography (CCTA) provides a non-invasive method to assess plaque characteristics and monitor CAD progression over time [11, 12]. In this study, we aimed to evaluate coronary plaques progression in a cohort of diabetic patients over 1 year after an ACS, and to assess differences between patients treated with GLP-1Ra and those receiving other antidiabetic agents.

## Methods

### Study design

The Coronary Artery Disease Progression in Patients With Acute Coronary Syndromes and Diabetes Mellitus (PROGRESSION) study is a prospective, single-center study designed to assess CAD progression following non-ST-elevation acute coronary syndrome (NSTE-ACS). Between March 2019 and December 2022, patients undergoing PCI for NSTE-ACS at Humanitas Research Hospital (Rozzano-Milan, Italy) were enrolled. Inclusion criteria encompassed adults aged 18–80 years who could provide informed consent and had at least one non-culprit lesion (NCL) with a diameter stenosis of ≥ 20% and < 50% at the index coronary angiography. Exclusion criteria were presentation with ST-segment elevation myocardial infarction, cardiogenic shock, suspected stent thrombosis, severe chronic kidney disease (estimated glomerular filtration rate [eGFR] < 30 mL/min), known hypersensitivity to aspirin or ticagrelor, childbearing potential, and a life expectancy of less than 1 year due to non-cardiac conditions. All participants were scheduled for CCTA and serial blood tests at baseline (after PCI during the index hospitalization) and at 1-year follow-up. Additionally, a clinical follow-up visit was performed at 1 year. The present prespecified analysis included patients with pre-existing or newly diagnosed T2D. The study was conducted in accordance with the principles of the Declaration of Helsinki, and all participants provided written informed consent before undergoing coronary angiography. Ethical approval was granted by the local Institutional Review Board, and the study was registered on ClinicalTrials.gov (NCT03890822).

### Study procedures

Patients were included after coronary angiography and PCI, performed following standard protocols. After PCI, all patients received antithrombotic therapy consisting of aspirin and P2Y_12_ inhibitors, along with optimal medical therapy for secondary cardiovascular prevention, glycemic control, and management of additional cardiovascular risk factors, such as blood pressure and low-density lipoprotein cholesterol, in line with contemporary guideline recommendations [9, 13, 14]. 

### Quantitative CCTA analysis

CCTA was performed using either retrospectively ECG-gated or prospectively ECG-triggered protocols on a 128-slice or a 256-slice scanner. All patients received premedication with nitroglycerin immediately before CCTA acquisition, and beta-blockers were administered when heart rate control was required. For all CCTAs, a quantitative coronary atherosclerosis assessment was conducted, following the guidelines of the Society of Cardiovascular Computed Tomography (SCCT) [15]. The analysis included coronary segments with diameter >2.0 mm, excluding stented segments and chronic total occlusions. Segments with poor image quality due to artifacts were also excluded. Atherosclerosis was defined as any tissue structure >1 mm² within the coronary artery wall that was distinct from surrounding epicardial tissue, fat, or the vessel lumen. Two cardiac radiologists further analyzed the datasets using a semi-automated quantitative plaque analysis software (QAngio CT, Medis, Leiden) to quantify and characterize atherosclerotic plaques in the coronary arteries and their branches. Plaque volume (PV, mm³) was calculated for each NCL, and plaque burden (PB) was normalized to vessel volume to account for variations in coronary anatomy. Total atheroma volume (TAV) was obtained by summing PVs across all NCL in a patient, while percent atheroma volume (PAV) was determined as the mean PB for each patient.

### Lipidomics, metabolomics and proteomics analysis

GLP-1Ra–treated patients and four randomly selected controls underwent comprehensive plasma metabolomic, lipidomic, and proteomic analysis at baseline and 1 year. Lipidomic analysis was conducted using a liquid-liquid extraction method with a chloroform/methanol (2:1, v/v) solvent system to efficiently isolate lipid species. Samples were vortexed, subjected to sonication at 4 °C for 30 min, and centrifuged at 3,000 rpm for 10 min. The lower lipid-containing phase was collected, dried under nitrogen, and resuspended in isopropyl alcohol/methanol (1:1, v/v) with the internal standard lysophosphatidylcholine (LPC 12:0). Ultra-performance liquid chromatography (UPLC) separation was carried out on an ACQUITY BEH C18 column using a gradient elution of ammonium formate-containing mobile phases. Mass spectrometry detection was performed using a Q Exactive MS (Thermo) in both electrospray ionization positive (ESI+) and negative (ESI-) modes, with optimized settings including a spray voltage of 3.0 kV (ESI+) and 3.2 kV (ESI-), a capillary temperature of 350 °C, and a sheath gas flow rate of 45 arb.

For metabolomics analysis, samples were processed by methanol-based extraction, followed by vortexing and sonication at 4 °C for 30 min to enhance metabolite solubilization. The samples were precipitated at -20 °C for 1 h, centrifuged at 12,000 rpm for 10 min at 4 °C, and the supernatant was collected for LC-MS analysis. Quality control (QC) samples were prepared by pooling aliquots from all samples to assess analytical stability and reproducibility. Separation was performed using a Vanquish Flex UPLC system with an ACQUITY HSS T3 column under a gradient of acetonitrile and 0.05% formic acid in water. Mass spectrometric analysis was conducted on a Q Exactive Plus MS (Thermo) with full scan (m/z 70–1050) and data-dependent MS2 (dd-MS2) modes, using high-energy collision dissociation (HCD) for fragmentation. Data were processed using Compound Discoverer 3.0 (Thermo) for peak alignment and metabolite identification.

Proteomic analysis involved the removal of high-abundance proteins such as human serum albumin (HSA) and immunoglobulin G (IgG) using a depletion spin column. After incubation with the resin at room temperature for 30 min, the flow-through containing the depleted protein fraction was collected and precipitated with ice-cold acetone overnight at -20 °C. The protein pellet was reconstituted in 50 mM ammonium bicarbonate, reduced with 10 mM dithiothreitol (DTT) at 56 °C for 1 h, alkylated with 20 mM iodoacetamide (IAA) in the dark for 1 h, and digested overnight at 37 °C using trypsin (enzyme-to-substrate ratio of 1:50). The digested peptides were desalted and analyzed by nano-LC-MS/MS using an Ultimate 3000 UHPLC system coupled to a high-resolution mass spectrometer. Peptide separation was performed on a PepMap C18 analytical column under a gradient elution of 0.1% formic acid in water and acetonitrile. MS data were acquired in Top 20 mode, with a full scan range of m/z 300–1650 at a resolution of 60,000, and MS/MS scans at a resolution of 15,000. The raw MS files were processed using MaxQuant (version 1.6.2.6) with database searches against a human protein database. Protein identification was based on fixed (carbamidomethylation of cysteines) and variable (oxidation of methionine) modifications, with a mass tolerance of 10 ppm for precursors and 0.02 Da for-fragment ions.

### Study endpoints

The primary endpoint was the change in PB from baseline to 1 year (∆PB = PB at 1-year – PB at baseline). Secondary endpoints included changes in PV (∆PV = PV at 1-year – PV at baseline) and its components (fibrous, fibrofatty volume, necrotic core and dense calcium volume), expressed as both absolute values (mm³) and percentages of PV, and the change in plaque thickness. Patient-level changes in PAV (∆PAV = PAV at 1 year – PAV at baseline) and TAV (∆TAV = TAV at 1 year – TAV at baseline) at 1 year were also evaluated.

Clinical outcomes assessed at 1-year follow-up included all-cause death, cardiovascular death, myocardial infarction (MI), stroke, definite or probable stent thrombosis (ST), target lesion revascularization, target vessel revascularization, and hospitalization for cardiovascular causes. All clinical endpoints were defined according to Academic Research Consortium-2 (ARC-2) criteria [15]. 

### Statistical analysis

Continuous variables are presented as mean ± standard deviation or median (interquartile range) and compared using Student’s t-test or Wilcoxon test based on data distribution. Categorical variables are shown as number (percentage) and compared using the chi-square test or the Fisher exact test, as appropriate. Statistical analyses for clinical, procedural and CCTA data were performed using Stata version 18.0 (StataCorp), with p-values < 0.05 considered as significant.

In lipidomics analysis, lipid species were identified using LipidSearch software (Thermo), with multivariate statistical analysis, including principal component analysis (PCA) and supervised regression models (PLS-DA, OPLS-DA), applied to identify significant lipid biomarkers based on variable importance in projection (VIP > 1.5), fold-change (|log2FC| >1), and statistical significance (*p* < 0.05). Statistical analyses for metabolomic data, including PCA, PLS-DA, and OPLS-DA, were performed in SIMCA-P (version 14.1), with potential biomarkers filtered based on VIP scores (> 1.5), statistical significance (*p* < 0.05), and fold-change values (|log2FC| >1). The quality of the model was evaluated using R² (variance explained) and Q² (predictive performance).

## Results

Twenty-eight patients with established or newly diagnosed T2D represented the present study cohort. GLP-1Ra therapy was prescribed at discharge in 7 patients (25%; dulaglutide: *n* = 5, semaglutide: *n* = 2), while 21 patients (75%) received other antidiabetic agents.

### Baseline clinical and procedural characteristics

Baseline characteristics are reported in Table 1. The mean age was 67.0 ± 10.5 years, and 21.4% of patients (*n* = 6) were women, with no significant differences between groups. The median BMI was 29.2 kg/m² (IQR: 26.2–32.3), and 35.7% (*n* = 10) were obese. Chronic kidney disease and prior MI were each present in 28.6% (*n* = 8). Median HbA1c was 6.5% (IQR: 6.2–7.3), with no significant difference between the GLP-1Ra (7.0% [IQR: 6.3–7.3]) and control groups (6.5% [IQR: 6.2–7.0]). 21.4% (*n* = 6) of patients had insulin-dependent T2D. Single-vessel CAD was observed in 75.0% (*n* = 21) of patients, with culprit vessel distribution differing between groups: the left circumflex artery was predominant in the GLP-1Ra group, while the left anterior descending and right coronary arteries were more common in controls. PCI with drug-eluting stents was performed in 92.9% (*n* = 26) of cases, with a median lesion length of 24.0 mm (IQR: 18.0–43.5) and a reference vessel diameter of 3.0 mm (IQR: 2.5–3.5). Antithrombotic, antidiabetic and other secondary prevention therapies at admission and discharge are listed in Supplementary Tables 1 and 2.

**Table 1:** Baseline clinical and procedural characteristics

	Overall (N = 28)	GLP-1Ra (N = 7)	No GLP-1Ra (N = 21)	p-value
Clinical characteristics				
Female sex	6 (21.4%)	3 (42.9%)	3 (14.3%)	0.11
Age (years), mean (SD)	67.0 (10.5)	64.3 (16.0)	68.0 (8.3)	0.43
BMI (Kg/m^2^), median (IQR)	29.2 (26.2, 32.3)	29.4 (25.4, 36.3)	29.1 (26.4, 31.4)	0.92
Hypertension	25 (89.3%)	7 (100.0%)	18 (85.7%)	0.29
Smoking	15 (53.6%)	3 (42.9%)	12 (57.1%)	0.51
LDL cholesterol (mg/dL), median (IQR)	80.0 (70.0, 109.0)	96.0 (80.0, 137.0)	76.0 (64.0, 107.0)	0.18
Family history of CAD	6 (21.4%)	1 (14.3%)	5 (23.8%)	0.59
eGFR (mL/min*1.73m^2^), mean (SD)	72.1 (19.8)	70.6 (21.2)	72.6 (19.8)	0.82
Chronic kidney disease	8 (28.6%)	5 (23.8%)	3 (42.9%)	0.33
Prior myocardial infarction	8 (28.6%)	3 (42.9%)	5 (23.8%)	0.33
Prior PCI	10 (35.7%)	3 (42.9%)	7 (33.3%)	0.65
LVEF (%), mean (SD)	52.9 (5.5)	55.0 (2.9)	52.2 (6.0)	0.26
Chronic obstructive pulmonary disease	2 (7.1%)	1 (14.3%)	1 (4.8%)	0.40
Prior stroke or TIA	2 (7.1%)	1 (14.3%)	1 (4.8%)	0.40
History of atrial fibrillation	3 (10.7%)	1 (14.3%)	2 (9.5%)	0.72
Peripheral artery disease	3 (10.7%)	0 (0.0%)	3 (14.3%)	0.29
High sensitivity CRP (mg/L), median (IQR)	0.3 (0.1, 0.8)	0.1 (0.1, 0.4)	0.4 (0.1, 1.0)	0.32
HbA1c (%), median (IQR)	6.5 (6.2, 7.3)	7.0 (6.3, 7.3)	6.5 (6.2, 7.0)	0.45
Insulin-dependent diabetes mellitus	6 (21.4%)	1 (14.3%)	5 (23.8%)	0.59
Clinical presentation				0.82
Unstable angina	11 (39.3%)	3 (42.9%)	8 (38.1%)	
NSTEMI	17 (60.7%)	4 (57.1%)	13 (61.9%)	
Time from symptom onset to PCI				0.004*
< 24 h	14 (50.0%)	2 (28.6%)	12 (57.1%)	
24–48 h	7 (25.0%)	0 (0.0%)	7 (33.3%)	
> 48 h	7 (25.0%)	5 (71.4%)	2 (9.5%)	
Peak high-sensitivity troponin I, median (IQR)	1692.5 (181.0, 3395.4)	2442.3 (976.3, 3981.7)	1524.0 (181.0, 2984.0)	0.48
Angiographic characteristics				
CAD extension				0.94
Single-vessel	21 (75.0%)	5 (71.4%)	16 (76.2%)	
Two-vessel	4 (14.3%)	1 (14.3%)	3 (14.3%)	
Three-vessel	3 (10.7%)	1 (14.3%)	2 (9.5%)	
Culprit vessel				0.036
Left main	2 (7.1%)	0 (0.0%)	2 (9.5%)	
Left anterior descending	9 (32.1%)	1 (14.3%)	8 (38.1%)	
Left circumflex	8 (28.6%)	5 (71.4%)	3 (14.3%)	
Right coronary artery	9 (32.1%)	1 (14.3%)	8 (38.1%)	
PCI device				0.40
Drug-eluting stent	26 (92.9%)	7 (100.0%)	19 (90.5%)	
Drug-coated balloon	2 (7.1%)	0 (0.0%)	2 (9.5%)	
Lesion length (mm), median (IQR)	24.0 (18.0, 43.5)	18.0 (15.0, 24.0)	28.0 (22.0, 45.0)	0.067
Reference vessel diameter (mm), median (IQR)	3.0 (2.5, 3.5)	2.8 (2.5, 3.0)	3.0 (2.8, 3.5)	0.097
Use of intravascular imaging	2 (7.1%)	0 (0.0%)	2 (9.5%)	0.40
Bifurcation involvement	3 (10.7%)	0 (0.0%)	3 (14.3%)	0.29
Other vessel PCI	7 (25.0%)	2 (28.6%)	5 (23.8%)	0.80

*BMI* body mass index, *CAD* coronary artery disease, *CRP* C-reactive protein, *eGFR* estimated glomerular filtration rate, *GLP1-ra* glucagon-like peptide-1 receptor agonist, *HbA1c* glycated hemoglobin, *IQR* interquartile range, *LDL* low-density lipoprotein, *LVEF* left ventricular ejection fraction, *NSTEMI* non-ST-elevation myocardial infarction, *PCI* percutaneous coronary intervention, *SD* standard deviation, *TIA* transient ischemic attack

^*^p < 0.05

#### Clinical outcomes at 1 year

Supplementary Table 3 details the 1-year clinical outcomes. One patient in the control group died from septic shock complicating pneumonia. One GLP-1Ra patient required target lesion revascularization. No additional adverse events occurred.

#### Quantitative CCTA analysis

**Fig. 1:**
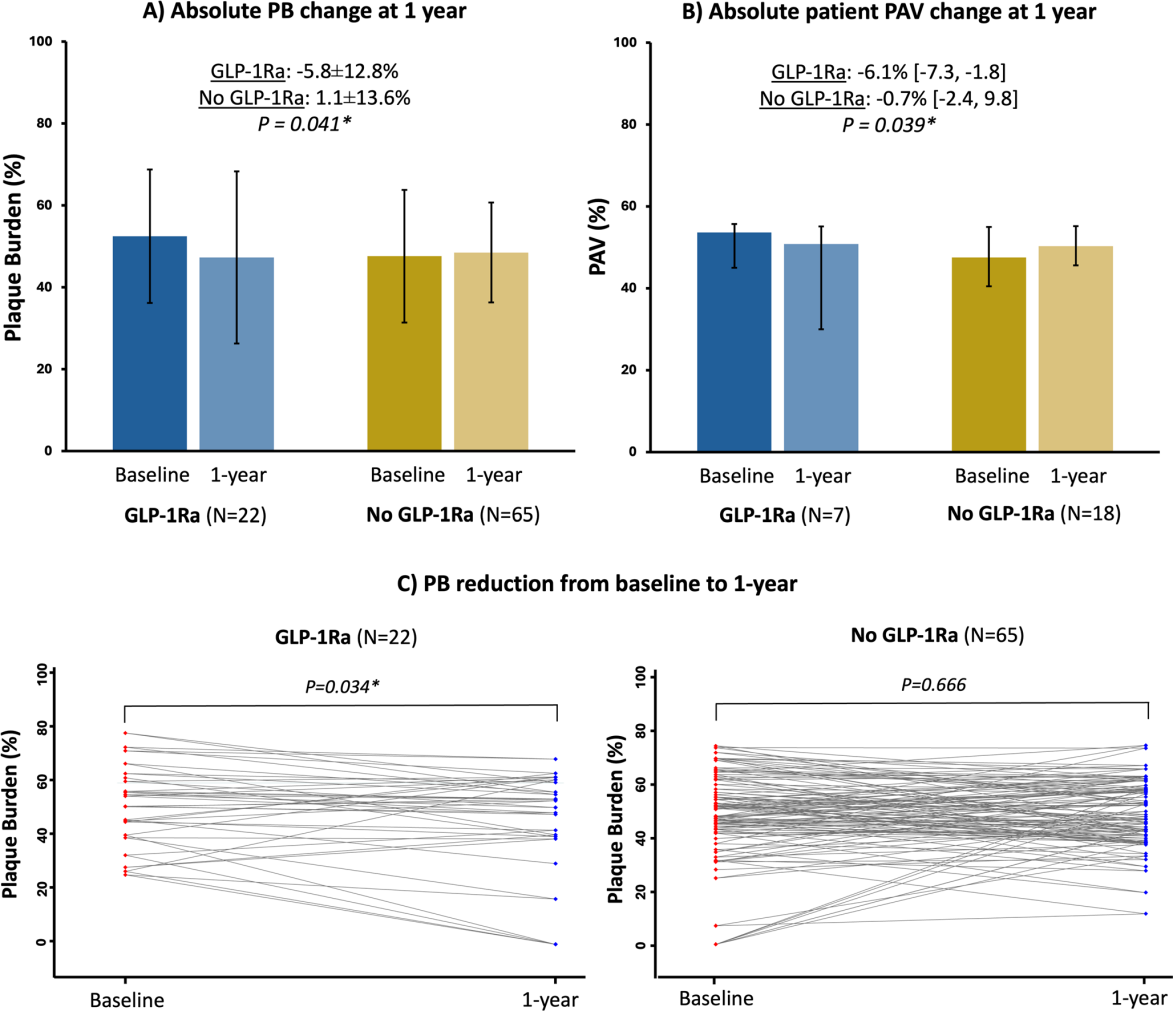
Changes in Plaque Burden and Percent Atheroma Volume at 1 year. Bar plots display the mean plaque burden (PB, panel **A**) and median percent atheroma volume (PAV, panel **B**) at baseline and at 12-month follow-up in the GLP-1Ra and control groups. Error bars represent standard deviations in panel **A** and interquartile ranges in panel **B**. The spaghetti plots (panel **C**) illustrate individual plaque burden changes from baseline to 1 year in the GLP-1Ra and control groups. *p-value < 0.05. *GLP-1Ra* glucagon-like peptide 1 receptor agonist, *PAV* percent atheroma volume, *PB* plaque burden

**Fig. 2:**
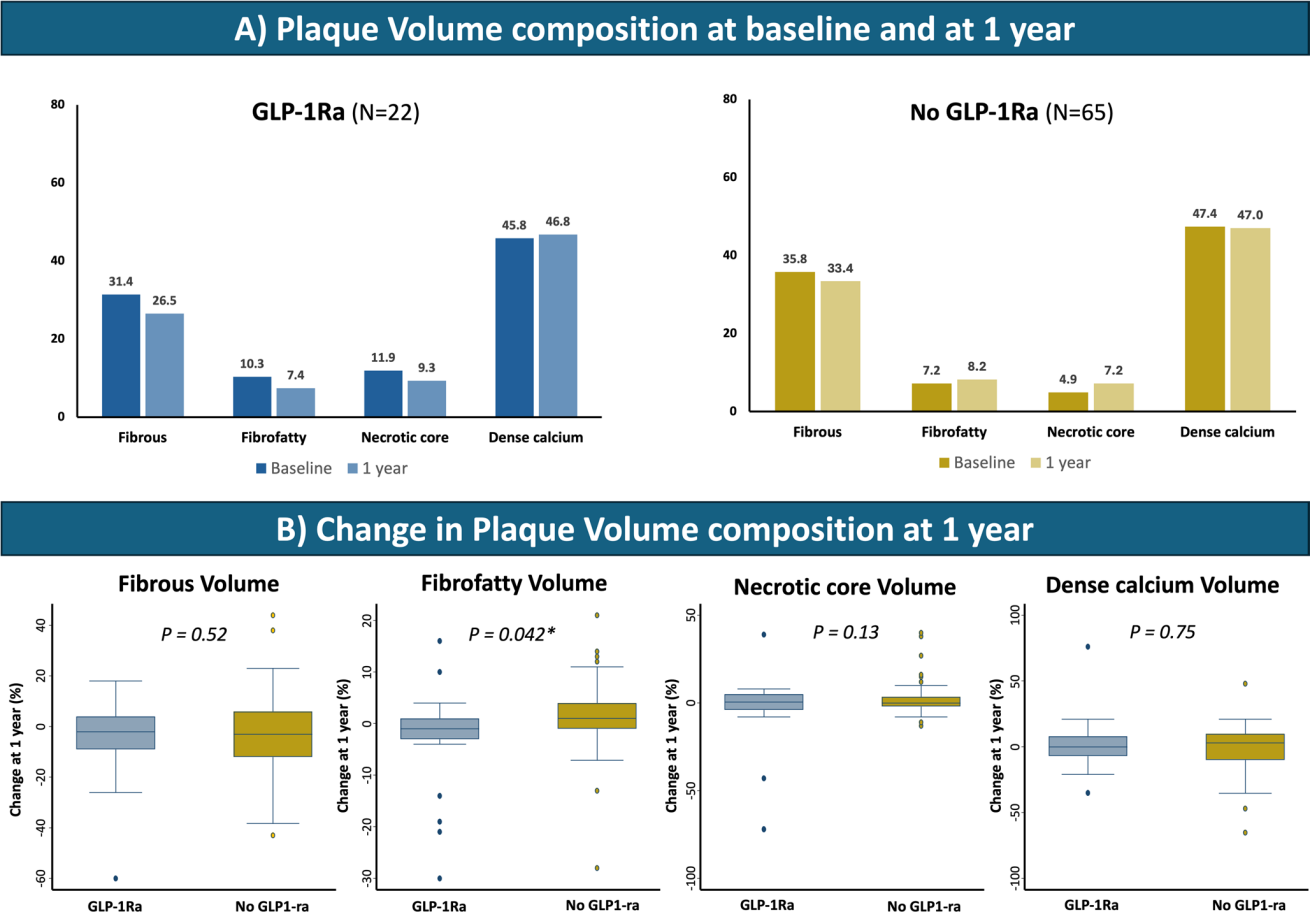
Changes in Plaque Volume Composition from baseline to 1 year. **A** Bar graphs display the percent composition of plaque volume per lesion (fibrous, fibro-fatty, necrotic core, and dense calcium) at baseline and after 1 year in the GLP-1Ra (N = 22) and control (No GLP-1Ra, N = 65) groups. **B** Box plots illustrate the percentage change in plaque volume composition over 1 year for each plaque component. *p-value < 0.05. *GLP-1Ra* glucagon-like peptide 1 receptor agonist

**Fig. 3:**
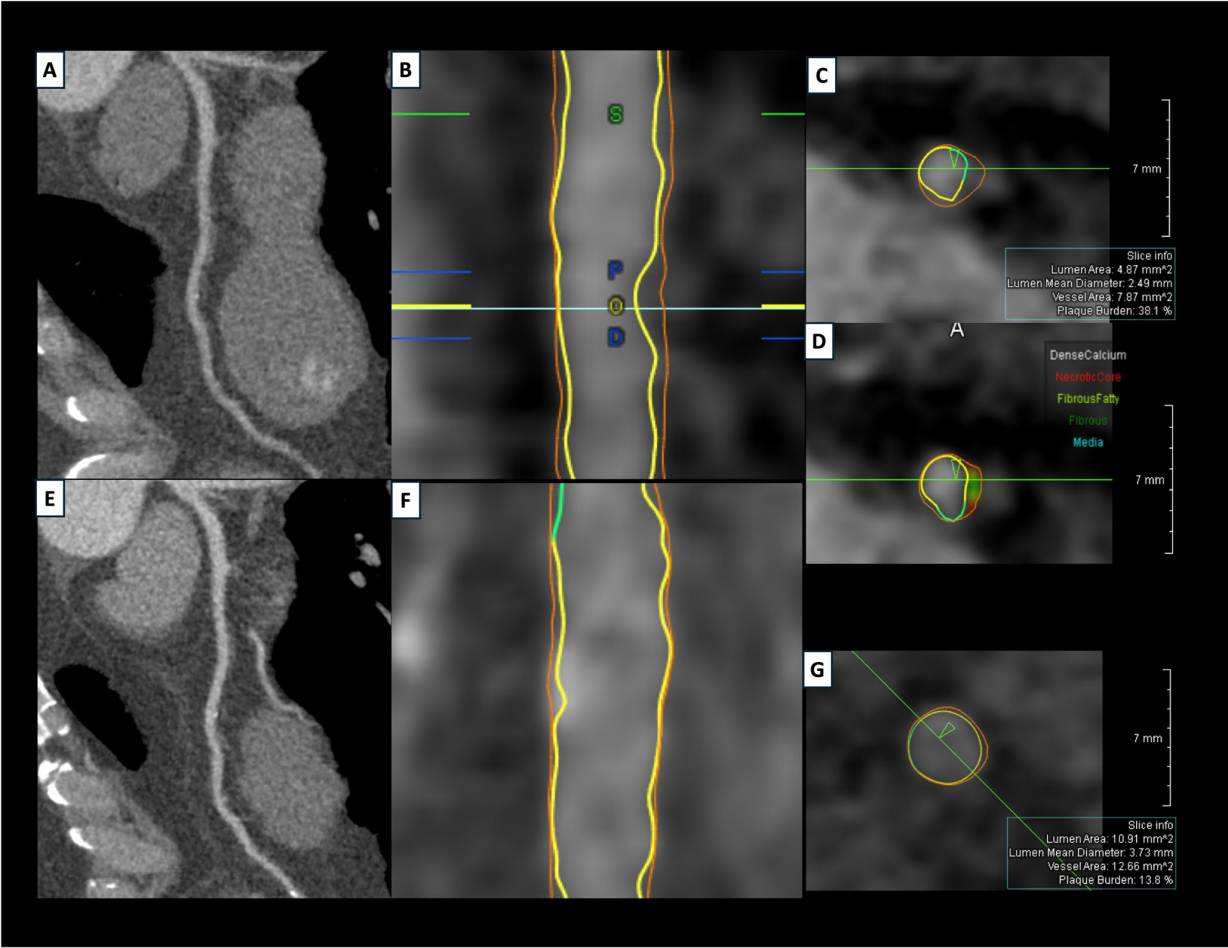
Case example of plaque regression in a patient treated with GLP-1Ra. A 46-year-old male patient presented with NSTE-ACS and underwent primary PCI on the right coronary artery. The initial CCTA (panels **A**–**D**) demonstrates a fibrous-fatty plaque in the mid-segment of the LAD, causing mild stenosis with a total plaque burden of 38.1%. At follow-up CCTA (panels **E**–**G**), advanced plaque analysis of the same LAD segment reveals minimal residual plaque (total plaque burden 13.8%) with near-complete restoration of the lumen area. *CCTA* coronary CT angiography, *LAD* left anterior descending artery

A total of 25 patients (7 GLP-1Ra, 18 control) underwent serial CCTA. Three control patients did not complete the 12-month scan (one death, two consent withdrawals). In total, 87 coronary plaques were analyzed (GLP-1Ra: *n* = 22, control: *n* = 65; Table 2). Baseline PV was significantly higher in GLP-1Ra patients (49.6 ± 53.4 mm³ vs. 27.7 ± 27.1 mm³, *p* = 0.015), while PB was comparable (52.5 ± 16.3% vs. 47.6 ± 16.2%, *p* = 0.22). At 1 year, GLP-1RA treatment was associated with significantly lower ΔPB (-5.8 ± 12.8% vs. 1.1 ± 13.6%, *p* = 0.041, Fig. 1A) and ΔPAV (-6.1% [IQR: -7.3, -1.8] vs. -0.7% [IQR: -2.4, 9.8], *p* = 0.039; Fig. 1B; Table 3). A significant PB regression was observed in the GLP-1Ra group (*p* = 0.034), but not in the control group (*p* = 0.666; Fig. 1C). At 1 year, ΔPV was 2.0 ± 15.0 mm³ (GLP-1Ra) vs. 5.7 ± 17.6 mm³ (control) (*p* = 0.38), while ΔTAV was numerically lower in GLP-1Ra group (0.7 mm³ [IQR: -2.5, 8.7] vs. 25.0 mm³ [IQR: 4.8, 39.7], *p* = 0.081; Tables 2 and 3). The percent decrease in fibrofatty volume was significantly greater in the GLP-1Ra group (-2.9 ± 10.1% vs. 1.0 ± 6.8%, *p* = 0.042; Fig. 2). A numerical reduction in plaque thickness was also noted (-0.3 ± 0.5 mm vs. 0.3 ± 2.0 mm, *p* = 0.20), with a greater proportion of plaques exhibiting thickness regression (81.8% vs. 50.0%, *p* = 0.009; Table 2). Figure 3 shows a case example of plaque regression on the left anterior descending coronary artery in a GLP-1Ra treated patient.

**Table 2:** Quantitative CCTA analysis per lesion

	GLP-1Ra (N = 22)	No GLP-1Ra (N = 65)	p-value
Plaque burden (%), mean (SD)			
Baseline	52.5 (16.3)	47.6 (16.2)	0.22
1-year	47.3 (21.0)	48.5 (12.2)	0.74
Absolute change	-5.8 (12.8)	1.1 (13.6)	0.041*
Plaque burden decrease, n(%)	12 (54.5%)	18 (28.1%)	0.025*
Plaque thickness (mm), mean (SD)			
Baseline	2.4 (0.9)	2.1 (0.7)	0.11
1-year	2.1 (0.9)	2.4 (1.9)	0.55
Absolute change	-0.3 (0.5)	0.3 (2.0)	0.20
Plaque thickness decrease, n(%)	18 (81.8%)	32 (50.0%)	0.009*
Plaque volume (mm^3^), mean (SD)			
Baseline	49.6 (53.4)	27.7 (27.1)	0.015*
1-year	51.5 (55.5)	33.4 (33.6)	0.072
Absolute change	2.0 (15.0)	5.7 (17.6)	0.38
Fibrous volume (mm^3^), mean (SD)			
Baseline	14.1 (16.0)	9.8 (8.2)	0.11
1-year	12.9 (14.4)	11.2 (11.6)	0.59
Absolute change	-1.2 (6.4)	1.5 (7.8)	0.15
Fibrous volume (%), mean (SD)			
Baseline	31.4 (12.4)	35.8 (15.9)	0.24
1-year	26.5 (14.2)	33.4 (12.7)	0.034*
Absolute change	-4.9 (16.2)	-2.4 (15.6)	0.52
Fibrofatty volume (mm^3^), mean (SD)			
Baseline	2.9 (2.9)	2.9 (8.4)	0.99
1-year	2.6 (3.0)	3.9 (8.7)	0.53
Absolute change	-0.2 (1.6)	1.0 (10.1)	0.57
Fibrofatty volume (%), mean (SD)			
Baseline	10.3 (9.3)	7.2 (6.1)	0.073
1-year	7.4 (7.5)	8.2 (6.1)	0.62
Absolute change	-2.9 (10.1)	1.0 (6.8)	0.042*
Necrotic core volume (mm^3^), mean (SD)			
Baseline	2.4 (2.4)	2.9 (11.7)	0.82
1-year	2.9 (3.9)	2.9 (9.7)	0.97
Absolute change	0.5 (2.9)	-0.0 (8.5)	0.79
Necrotic core volume (%), mean (SD)			
Baseline	11.9 (17.6)	4.9 (7.9)	0.014*
1-year	9.3 (13.5)	7.2 (11.7)	0.49
Absolute change	-2.6 (20.5)	2.4 (9.1)	0.13
Dense calcium volume, mean (SD)			
Baseline	26.6 (33.9)	14.2 (14.0)	0.018*
1-year	28.0 (35.9)	15.5 (15.3)	0.025*
Absolute change	1.4 (9.2)	1.3 (9.5)	0.95
Dense calcium volume (%), mean (SD)			
Baseline	45.8 (25.0)	47.4 (21.6)	0.77
1-year	46.8 (27.2)	47.0 (19.0)	0.98
Absolute change	1.0 (22.0)	-0.4 (17.5)	0.75

Abbreviations as in Table 1

*p < 0.05

**Table 3:** Quantitative CCTA analysis per patient

	GLP-1Ra (N = 7)	No GLP-1Ra (N = 18)	p-value
Percent atheroma volume (%), median (IQR)			
Baseline	53.6 (45.0, 55.7)	47.5 (40.5, 55.0)	0.47
1-year	50.8 (30.0, 55.1)	50.3 (45.6, 55.2)	0.68
Absolute change	-6.1 (-7.3, -1.8)	-0.7 (-2.4, 9.8)	0.039*
Total atheroma volume (mm^3^), median (IQR)			
Baseline	92.3 (15.3, 143.1)	125.9 (67.3, 139.5)	0.39
1-year	88.9 (16.5, 186.4)	132.7 (81.4, 183.2)	0.29
Absolute change	0.7 (-2.5, 8.7)	25.0 (4.8, 39.7)	0.081

Abbreviations as in Table 1

*p < 0.05

#### Lipidomics, metabolomics and proteomics analysis

At baseline, the two treatment groups exhibited distinct lipidomic and metabolomic profiles, with differences in glycerophosphocholine, monoacylglycerol (MAG), and triradylglycerols in lipidomics (Supplementary Fig. 1A) and tryptophan, starch, and sucrose metabolism in metabolomics (Supplementary Fig. 1B). Proteomic pathway enrichment highlighted extracellular vesicle dynamics, complement activation, and epithelial structure organization (Supplementary Fig. 1C).

**Fig. 4:**
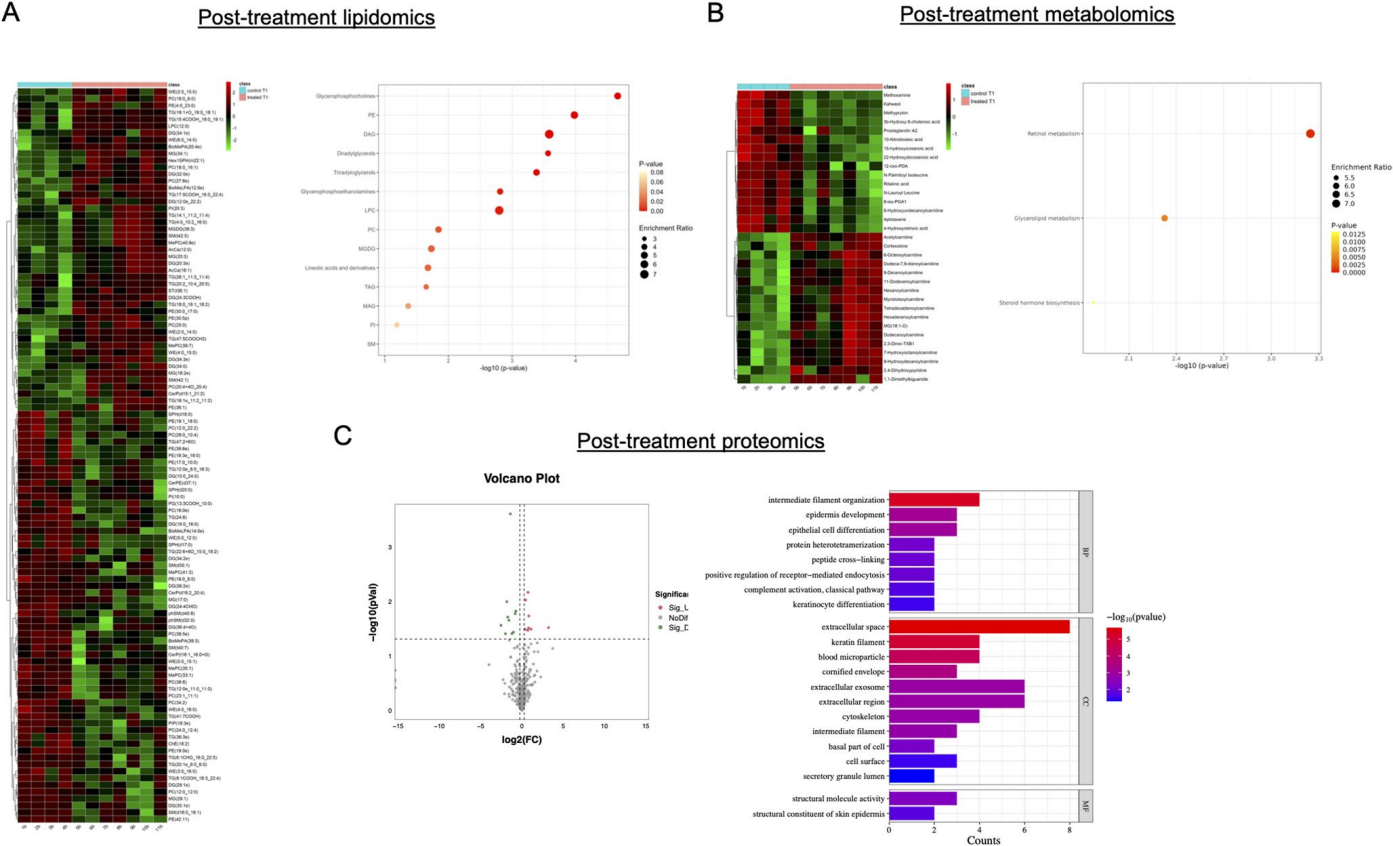
Differential Profiling of Lipidomic, Metabolomic, and Proteomic Responses at 1 year after treatment initiation. (Panel **A**) Hierarchical cluster analysis of lipidomics data post-treatment (T1) comparing control and treated groups (left panel). Pathway enrichment analysis of lipids is displayed on the right, highlighting significant lipid pathways altered by treatment. (Panel **B**) Hierarchical cluster analysis of metabolomics data post-treatment (T1) for control and treated groups (left panel). The right panel shows pathway enrichment analysis of metabolites, indicating key metabolic pathways affected by the treatment. (Panel **C**) Volcano plot depicting differentially expressed proteins between control and treated groups following semaglutide administration (left panel). The right panel presents the distribution of differentially expressed proteins categorized by Gene Ontology (GO) terms, emphasizing biological processes impacted by treatment

Lipidomic changes indicated a metabolic shift toward increased lipid utilization and improved energy efficiency in GLP-1Ra treated patients (Fig. 4A). In particular, we observed a decrease in MAG and triacylglycerol (TAG) and an increase in phosphatidylethanolamine (PE) and diacylglycerols (DAG). The metabolomic profile showed a shift from carbohydrate metabolism (tryptophan, starch, and sucrose pathways) toward lipid metabolism and hormone regulation among GLP-1Ra treated patients, including retinol metabolism, glycerolipid metabolism, and steroid hormone biosynthesis (Fig. 4B). Patients in the GLP-1Ra group also displayed significant changes in plasma proteomics as compared to their controls, particularly in proteins associated with immune regulation, endothelial function, and structural integrity (Fig. 4C). Complement C1q subcomponent subunit C, a key immune regulator, was differentially expressed, alongside keratin cytoskeletal components, Intercellular Adhesion Molecule 1 (ICAM-1), Neuropilin-1, L-selectin, and Endoplasmic Reticulum Chaperone BiP. Pathway analysis highlighted extracellular organization, keratinocyte differentiation, complement activation, and endothelial function, with enrichment in pathways related to angiogenesis (GO:0045766), receptor-mediated endocytosis (GO:0048260), and structural integrity of the epidermis and vasculature.

## Discussion

The present study aimed to assess coronary plaques progression during one year after an ACS in diabetic patients treated with GLP-1Ra. The main findings are as follows:

1. Despite similar baseline PB, patients receiving GLP-1Ra experienced significantly greater regression of atherosclerotic PB on NCLs, accompanied by a reduction in PAV.
2. GLP-1Ra treatment was also associated with changes in plaque morphology, including a greater reduction in fibrofatty volume and plaque thickness.
3. In the same patient cohort, a metabolic shift from carbohydrate reliance toward more efficient lipid utilization was observed, along with modulation of immune and endothelial responses, which may contribute to vascular stabilization and inhibition of atherosclerosis progression in diabetic ACS patients.

GLP-1Ra are glucose-lowering agents that activate GLP-1R, enhancing insulin secretion in a glucose-dependent manner [16, 17]. Evidence from eight landmark randomized clinical trials (RCTs) evaluating various GLP-1Ra in patients with T2D at high cardiovascular risk has consistently demonstrated their cardioprotective benefits, including reductions in cardiac mortality, MI, and stroke [10]. Notably, GLP-1Ra appear more effective in reducing atherosclerotic events compared to SGLT2 inhibitors, whose primary benefit lies in reducing heart failure hospitalizations and related mortality [18–20]. Beyond glycemic control, GLP-1Ra have been associated with several pleiotropic effects, including blood pressure reduction through improved endothelium-dependent vasodilation, weight loss, enhanced renal function via increased natriuresis and reduced albuminuria, as well as anti-inflammatory and vascular protective effects, including increased nitric oxide production and reduced oxidative stress [21–24]. 

A key consequence of these mechanisms may be protection against atherosclerosis progression and promotion of plaque regression, attributable not only to improved control of cardiovascular risk factors but also to reduced oxidative stress and decreased formation of proinflammatory macrophages and foam cells [22]. A direct anti-atherosclerotic effect of GLP-1Ra has so far been mainly evaluated in preclinical studies. In diabetic apolipoprotein-E (ApoE) knockout mice, early administration of dulaglutide was shown to reduce plaque area in the aortic arch [12]. Similarly, in a study using ApoE-deficient and LDL-deficient mice, semaglutide and liraglutide were found to decrease aortic plaque lesion area independent of body weight reduction and cholesterol lowering [25]. In a small human study involving 62 T2D patients, treatment with liraglutide was associated with reduced carotid intima-media thickness at four months [26]. 

The present analysis represents the first evaluation of coronary plaque regression with GLP-1Ra following ACS. In this setting, GLP-1Ra treatment appeared to promote plaque stabilization, leading to both a reduction in overall atherosclerotic PB and a decrease in fibrofatty volume. Notably, PB and fibrofatty volume have been identified as strong predictors of plaque rupture in ACS [27]. Consistently, TAV remained stable from baseline to the one-year follow-up in patients treated with GLP-1Ra, whereas a numerical increase was observed in those receiving other antidiabetic therapies. Importantly, diabetic ACS patients, unlike those with stable atherosclerotic vascular disease, face a heightened risk of plaque instability, with a greater proportion of vulnerable plaques, which contributes to an increased likelihood of CAD progression on NCLs [28–30]. This distinction may explain the discrepancy between our findings and those of the recently presented STOP (Semaglutide Treatment Effect On Coronary Atherosclerosis Progression In Diabetes) trial, which assessed non-calcified PV progression via CCTA in 140 T2D patients with chronic atherosclerotic disease and failed to demonstrate the superiority of semaglutide over placebo [31]. 

To further support and validate the findings of the quantitative CCTA analysis, we also conducted a comprehensive lipidomic, metabolomic, and proteomic assessment in our study cohort, yielding two key insights. First, GLP-1Ra influenced lipid homeostasis by promoting fat breakdown, energy balance, and metabolic adaptation. The observed reduction in MAG and TAG aligns with the known effects of GLP-1Ra in decreasing fat accumulation and enhancing lipolysis, contributing to weight loss [32, 33]. The increase in DAG, a critical lipid metabolism intermediate, suggests enhanced lipid mobilization rather than storage, reinforcing the shift from carbohydrate dependence to efficient lipid utilization [34]. Consistently, a metabolic transition indicating decreased reliance on carbohydrate metabolism was observed, likely due to GLP-1Ra’s ability to enhance insulin secretion, suppress glucagon, and improve glucose homeostasis [35, 36]. Additionally, the increase in steroid hormone biosynthesis may reflect potential modulation of inflammatory or endocrine functions, and a direct effect on T lymphocytes as recently demonstrated [37–39]. Second, proteomic analyses revealed shifts in complement activation, cytoskeletal organization, and cell adhesion pathways, supporting the hypothesis that GLP-1Ra exert anti-inflammatory and vasoprotective effects, potentially contributing to reduced CAD progression following ACS. These coordinated metabolic and proteomic shifts may ultimately favor plaque regression by reducing lipid accumulation within the arterial wall, dampening inflammatory activation, and improving endothelial repair and stability.

Although our small cohort was underpowered to assess the impact of these quantitative atherosclerotic changes on clinical outcomes, these mechanisms may at least partially explain the treatment effects associated with GLP-1Ra in large-scale RCTs. Notably, both a pooled analysis of SUSTAIN-6 and PIONEER and the REWIND trial, evaluating semaglutide and dulaglutide, respectively, in diabetic patients at high cardiovascular risk, demonstrated a consistent reduction in cardiovascular events, particularly in those with established atherosclerotic disease [40, 41]. However, dedicated studies investigating currently recommended GLP-1Ra (semaglutide, dulaglutide, and liraglutide) in ACS patients are still lacking, and the only trial exclusively enrolling patients with recent ACS - the ELIXA trial - failed to demonstrate the superiority of lixisenatide over placebo [42]. 

### Limitations

First, as an observational study, our findings should be considered hypothesis-generating rather than definitive. Second, given the small sample size, the study was underpowered to assess the impact of quantitative CCTA parameters on clinical outcomes, as well as to detect differences in event rates between the GLP-1Ra and control groups. Third, we did not perform multivariable-adjusted analyses due to the risk of statistical overfitting. However, potential bias was minimized by the comparable baseline clinical characteristics, CCTA parameters, and discharge medications between study groups. Finally, semaglutide and dulaglutide were the only GLP-1RA in this study, and a drug-specific rather than class effect cannot be excluded.

## Conclusions

In patients with T2D and ACS, treatment with GLP-1Ra was associated with a regression of the atherosclerotic plaque burden over 1 year. Additionally, GLP-1Ra treatment was linked to a metabolic shift toward lipid utilization, modulation of the immune response, and improved endothelial function, which may contribute to its favorable effect on CAD regression. Further RCTs are needed to evaluate the role of GLP-1Ra in limiting CAD progression and improving cardiovascular outcomes after ACS.

## Supplementary Information

Below is the link to the electronic supplementary material.

Supplementary Material 1

## Data Availability

The datasets used and/or analysed during the current study are available from the corresponding author on reasonable request.
