# Supplementary material for: GLP-1 receptor agonists and coronary plaques regression in diabetic patients after acute coronary syndromes
Source: Acta Diabetol. 2025 Nov 4;63(2):179–91. doi: 10.1007/s00592-025-02606-z (PMC12957045; doi:10.1007/s00592-025-02606-z)
Supplement: Supplementary file 1 — Supplementary Material 1 [file 592_2025_2606_MOESM1_ESM.docx]

**Supplementary Materials**

**Manuscript title:** GLP-1 Receptor Agonists and Coronary Plaques Regression in Diabetic Patients after Acute Coronary Syndromes

Table of contents

[Supplementary Table 1. Admission medications 2](#_Toc211187564)

[Supplementary Table 2. Discharge medications 3](#_Toc211187565)

[Supplementary Table 3. Clinical outcomes at 12 months 4](#_Toc211187566)

[Supplementary Figure 1. Differential profiling of pretreatment lipidomics, metabolomics, and proteomics in plasma 5](#_Toc211187567)

# Supplementary Table 1. Admission medications

|  | **Overall**  **(N=28)** | **GLP-1Ra**  **(N=7)** | **No GLP-1Ra**  **(N=21)** | **p-value** |
| --- | --- | --- | --- | --- |
| Aspirin | 11 (39.3%) | 3 (42.9%) | 8 (38.1%) | 0.82 |
| P2Y_12_ inhibitor | 5 (17.9%) | 0 (0.0%) | 5 (23.8%) | 0.15 |
| Oral anticoagulant | 3 (10.7%) | 1 (14.3%) | 2 (9.5%) | 0.72 |
| Statin | 15 (53.6%) | 4 (57.1%) | 11 (52.4%) | 0.83 |
| High-intensity statin | 8 (28.6%) | 3 (42.9%) | 5 (23.8%) | 0.33 |
| Beta-blocker | 15 (53.6%) | 4 (57.1%) | 11 (52.4%) | 0.83 |
| ACE inhibitor or ARB | 14 (50.0%) | 3 (42.9%) | 11 (52.4%) | 0.66 |
| Insulin | 6 (21.4%) | 1 (14.3%) | 5 (23.8%) | 0.59 |
| Metformin | 6 (21.4%) | 2 (28.6%) | 4 (19.0%) | 0.59 |
| SGLT2 inhibitors | 2 (7.1%) | 0 (0.0%) | 2 (9.5%) | 0.40 |
| GLP-1Ra | 3 (10.7%) | 2 (28.6%) | 1 (4.8%) | 0.078 |

Abbreviations: ACE, angiotensin-converting enzyme; ARB, angiotensin receptor blocker; GLP-1Ra, glucagon-like peptide-1 receptor agonist; SGLT2, sodium-glucose cotransporter-2.

# Supplementary Table 2. Discharge medications

|  | **Overall**  **(N=28)** | **GLP-1Ra**  **(N=7)** | **No GLP-1Ra**  **(N=21)** | **p-value** |
| --- | --- | --- | --- | --- |
| Aspirin | 25 (89.3%) | 7 (100.0%) | 18 (85.7%) | 0.62 |
| P2Y_12_ inhibitor |  |  |  | 0.69 |
| Ticagrelor | 18 (64.3%) | 4 (57.1%) | 14 (66.7%) |  |
| Clopidogrel | 9 (32.1%) | 3 (42.9%) | 6 (28.6%) |  |
| Prasugrel | 1 (3.6%) | 0 (0.0%) | 1 (4.8%) |  |
| Oral anticoagulant | 3 (10.7%) | 1 (14.3%) | 2 (9.5%) | 0.72 |
| Statin | 27 (96.4%) | 7 (100.0%) | 20 (95.2%) | 0.56 |
| High-intensity statin | 23 (82.1%) | 6 (85.7%) | 17 (81.0%) | 0.78 |
| Beta-blocker | 26 (92.9%) | 6 (85.7%) | 20 (95.2%) | 0.40 |
| ACE inhibitor or ARB | 19 (67.9%) | 4 (57.1%) | 15 (71.4%) | 0.48 |
| Insulin | 6 (21.4%) | 1 (14.3%) | 5 (23.8%) | 0.59 |
| Metformin | 16 (57.1%) | 6 (85.7%) | 10 (47.6%) | 0.078 |
| SGLT2 inhibitors | 4 (14.3%) | 1 (14.3%) | 3 (14.3%) | 1.00 |
| DPP4 inhibitors | 1 (3.6%) | 0 (0.0%) | 1 (4.8%) | 0.56 |

Abbreviations: ACE, angiotensin-converting enzyme; ARB, angiotensin receptor blocker; DPP4, dipeptidyl peptidase-4; GLP-1Ra, glucagon-like peptide-1 receptor agonist; SGLT2, sodium-glucose cotransporter-2.

# Supplementary Table 3. Clinical outcomes at 12 months

|  | **Overall**  **(N=28)** | **GLP-1Ra**  **(N=7)** | **No GLP-1Ra**  **(N=21)** | **p-value** |
| --- | --- | --- | --- | --- |
| All-cause death | 1 (3.7%) | 0 (0.0%) | 1 (5.0%) | 0.55 |
| Cardiovascular death | 0 (0.0%) | 0 (0.0%) | 0 (0.0%) | - |
| Myocardial infarction | 0 (0.0%) | 0 (0.0%) | 0 (0.0%) | - |
| Stent thrombosis | 0 (0.0%) | 0 (0.0%) | 0 (0.0%) | - |
| Stroke | 0 (0.0%) | 0 (0.0%) | 0 (0.0%) | - |
| Target lesion revascularization | 1 (3.7%) | 1 (14.3%) | 0 (0.0%) | 0.085 |
| Target vessel revascularization | 1 (3.7%) | 1 (14.3%) | 0 (0.0%) | 0.085 |
| Hospitalization for cardiovascular causes | 1 (3.7%) | 1 (14.3%) | 0 (0.0%) | 0.085 |

Abbreviations as in previous Tables.

# Supplementary Figure 1. Differential profiling of pretreatment lipidomics, metabolomics, and proteomics in plasma

(A) Hierarchical cluster analysis of lipidomics data at pre-treatment (T0) comparing control and treated groups (left panel). Pathway enrichment analysis of lipids (right panel) highlights key lipid pathways significantly altered by the treatment. (B) Hierarchical cluster analysis of metabolomics data at pre-treatment (T0) for both control and treated groups (left panel). The right panel displays pathway enrichment analysis of metabolites, revealing the metabolic pathways that are most affected by the treatment. (C) Volcano plot illustrating differentially expressed proteins between control and treated groups prior to semaglutide administration (left panel). The right panel shows the distribution of differentially expressed proteins, categorized by Gene Ontology (GO) terms, underscoring the biological processes that are influenced by the treatment.
